# Draft Genome Sequences of the Xylose-Fermenting Yeast *Scheffersomyces shehatae* NBRC 1983^T^ and a Thermotolerant Isolate of *S. shehatae* ATY839 (JCM 18690)

**DOI:** 10.1128/genomeA.00347-17

**Published:** 2017-05-18

**Authors:** Natsumi Okada, Ayumi Tanimura, Hideki Hirakawa, Masako Takashima, Jun Ogawa, Jun Shima

**Affiliations:** a Division of Applied Life Sciences, Graduate School of Agriculture, Kyoto University, Kyoto, Japan; b Department of Technology Development, Kazusa DNA Research Institute, Chiba, Japan; c Japan Collection of Microorganisms, RIKEN BioResource Center, Ibaraki, Japan; d Faculty of Agriculture, Ryukoku University, Shiga, Japan

## Abstract

Draft genome sequences of the type strain (NBRC 1983) and a thermotolerant isolate (ATY839) of the xylose-fermenting yeast *Scheffersomyces shehatae* were determined. The genome sizes and presumed open reading frames were highly similar between strains NBRC 1983^T^ and ATY839.

## GENOME ANNOUNCEMENT

*Scheffersomyces shehatae* is one of the most common xylose-fermenting yeasts (1, 2). *S. shehatae* ATY839 (available from the Japan Collection of Microorganisms as JCM 18690) was isolated from soil at Kyoto University (3). Based on 26S rRNA gene analysis, this strain and *S. shehatae* CBS 4704 showed a nucleotide identity of 99.5% on average, suggesting they are the same species. *S. shehatae* ATY839 was more thermotolerant than other xylose-fermenting strains and produced ethanol at 37°C (3); therefore, ATY839 would be suitable for producing ethanol from lignocellulosic sugars through simultaneous saccharification and fermentation (SSF). High-temperature SSF with a thermotolerant yeast reduces contamination risk and offers stable fermentation even in tropical countries. We also found that this strain is promising for ethanol production from starch through consolidated bioprocessing (4). To obtain genomic information related to genes, including sugar transporters for biotechnological applications, we determined the draft genome sequences of the type strain of *S. shehatae* (NBRC 1983) and ATY839.

The single-end reads obtained by Roche 454 GS FLX+ and paired-end reads obtained by Illumina MiSeq platforms were assembled using Newbler version 2.3 (Roche Diagnostics). The total lengths of the genome sequences of NBRC 1983^T^ and ATY839 were 16,750,582 bp (444 contigs in 13 scaffolds, with a maximum length of 3,044,122 bp and *N*_50_ length of 2,182,181 bp) and 17,147,083 bp (779 contigs in 10 scaffolds, with a maximum length of 3,488,706 bp and *N*_50_ length of 2,759,450 bp), with 40.9% and 40.2% G+C content, respectively. Gene prediction was performed using AUGUSTUS (5) with a training set of *Saccharomyces cerevisiae* S288C. The functions of the predicted 6,162 open reading frames (ORFs) were inferred by using BLAST searches against the NCBI NR database (https://blast.ncbi.nlm.nih.gov). By conducting reciprocal BLAST searches, 5,782 ORFs were found in common between the NBRC 1983^T^ and ATY839 sequences assembled in this study. On the other hand, 181 ORFs were uniquely found in ATY839 and may be responsible for its strain-specific characteristics, including xylose fermentation capability at elevated temperature.

Based on a BLAST search against the euKaryotic clusters of Orthologous Groups (KOG) database, 1,188 ORFs were assigned to the metabolism category. In addition, 1,357 ORFs were assigned to activities involved in cellular processes and signaling, including defense mechanisms. By conducting functional annotation using an InterProScan search against the InterPro database (6), 25 genes coding for heat shock proteins were found. These proteins promote inducible thermotolerance in closely related microorganisms and are expected to be crucial for survival at high temperature (7). This study revealed genes related to xylose fermentation and the molecular basis of the traits that have made *S. shehatae* strains, such as ATY839 attractive for industrial applications. The sequence reported here would be useful as a genetic resource for engineering metabolism in the top-fermenting yeast *Saccharomyces cerevisiae* to improve its ability to ferment xylose to ethanol. Further studies on this comparative analysis are under way.

### Accession number(s).

The genome sequences of *S. shehatae* ATY839 and NBRC 1983^T^ are available in DDBJ/EMBL/GenBank databases under the accession numbers BDMO01000001 to BDMO01000010 and BDMP01000001 to BDMP01000013, respectively.
